# Two Influential Primate Classifications Logically Aligned

**DOI:** 10.1093/sysbio/syw023

**Published:** 2016-03-22

**Authors:** Nico M. Franz, Naomi M. Pier, Deeann M. Reeder, Mingmin Chen, Shizhuo Yu, Parisa Kianmajd, Shawn Bowers, Bertram Ludäscher

**Affiliations:** ^1^School of Life Sciences, PO Box 874501, Arizona State University, Tempe, AZ 85287, USA;; ^2^Department of Biology, Bucknell University, 1 Dent Drive, Lewisburg, PA 17837, USA;; ^3^Department of Computer Science, 2063 Kemper Hall, 1 Shields Avenue, University of California at Davis, CA 95616, USA;; ^4^Department of Computer Science, 502 East Boone Avenue, AD Box 26, Gonzaga University, Spokane, WA 99258, USA;; ^5^Gradate School of Library and Information Science, 510 East Daniel Street, University of Illinois at Urbana-Champaign, Champaign, IL 61820,

**Keywords:** Alignment, classification, concept taxonomy, logic, ontology, Primates, reasoning, Region Connection Calculus

## Abstract

Classifications and phylogenies of perceived natural entities change in the light of new evidence. Taxonomic changes, translated into Code-compliant names, frequently lead to name:meaning dissociations across succeeding treatments. Classification standards such as the *Mammal Species of the World* (MSW) may experience significant levels of taxonomic change from one edition to the next, with potential costs to long-term, large-scale information integration. This circumstance challenges the biodiversity and phylogenetic data communities to express taxonomic congruence and incongruence in ways that both humans and machines can process, that is, to logically represent taxonomic alignments across multiple classifications. We demonstrate that such alignments are feasible for two classifications of primates corresponding to the second and third MSW editions. Our approach has three main components: (i) use of taxonomic concept labels, that is name sec. author (where sec. means *according to*), to assemble each concept hierarchy separately via parent/child relationships; (ii) articulation of select concepts across the two hierarchies with user-provided Region Connection Calculus (RCC-5) relationships; and (iii) the use of an Answer Set Programming toolkit to infer and visualize logically consistent alignments of these input constraints. Our use case entails the Primates sec. Groves (1993; MSW2–317 taxonomic concepts; 233 at the species level) and Primates sec. Groves (2005; MSW3–483 taxonomic concepts; 376 at the species level). Using 402 RCC-5 input articulations, the reasoning process yields a single, consistent alignment and 153,111 Maximally Informative Relations that constitute a comprehensive meaning resolution map for every concept pair in the Primates sec. MSW2/MSW3. The complete alignment, and various partitions thereof, facilitate quantitative analyses of name:meaning dissociation, revealing that nearly one in three taxonomic names are not reliable across treatments—in the sense of the same name identifying congruent taxonomic meanings. The RCC-5 alignment approach is potentially widely applicable in systematics and can achieve scalable, precise resolution of semantically evolving name usages in synthetic, next-generation biodiversity, and phylogeny data platforms.

Primatologists on the whole don’t understand taxonomy, but they need to, and, in the main, they want to.

*(Groves 2001a:vii)*

Human classifications of perceived natural groups change in the light of new evidence. Over time these changes can affect the validity of taxonomic names and stability of their meanings. Users must keep track of name:meaning (read: “name-to-meaning”) relationship updates to communicate reliably about perceived organismal groups and retain the ability to integrate information across multiple incongruent classifications. Due to a general trend in biology toward generating synthetic data sets that may reflect heterogeneous taxonomic perspectives (e.g., Hinchcliff *et al.* 2015), the challenge of reconciling evolving taxonomies is becoming increasingly relevant (Franz *et al.* 2008). Few tools are designed specifically to model conflicts and ambiguities in name:meaning relationships that result from taxonomic and phylogenetic advancement.

More than 250 years since Linnaeus’ *Systema Naturae*, mammal classifications continue to change at both lower and higher taxonomic levels (e.g., Asher and Helgen 2010; Heller *et al.* 2013; Zachos *et al.* 2013; Cotterill *et al.* 2014). Reasons for such change are manifold, including the application of alternative species concepts or recognition of new phylogenetic information. Although a great number of changes in the mammal tree of life are also mirrored in emendations of mammalian nomenclature, the tracking of taxonomic changes through the type-centric Linnaean naming system is not perfect (Franz 2005; Kennedy *et al.* 2005; Witteveen 2015; Franz *et al.* 2016; Remsen 2016).

Why model evolving name:meaning relationships in systematics? Many systematists find it desirable that taxonomic name usages are not in conflict with contemporary phylogenetic inferences. We rely on the former (names) to integrate the latter (classifications, phylogenies) over time, and thereby build a long-term semantic foundation for refining evolutionary knowledge. The many-to-many relationships that frequently develop between taxonomic names and their meanings challenge this objective. At least two apparent solutions exist. The first is to promote stability and unity in classification, for instance through the adoption of community-wide standards that endorse specific configurations of valid names, synonyms, and taxonomic circumscriptions (Scoble 2004). In the present context, the *Mammal Species of the World* (henceforth: MSW) editions are standard references for mammalian classifications that aim to unify name:meaning usages at the global scale (Honacki *et al.* 1982; Wilson and Reeder 1993, 2005). However, even standards experience an evolution of changing taxonomic names and/or meanings from one edition to the next (Patterson 1994; Reeder *et al.* 2007; Solari and Baker 2007). This is exemplified by the family-level name Cebidae Bonaparte 1831, whose circumscription varies significantly across MSW editions and other treatments (Groves 1993, 2001a, 2005; Rylands and Mittermeier 2009). In short, standards can mitigate the challenges inherent in name-based data integration—up to a point. They cannot eliminate systemic limitations incurred by using names and nomenclatural relationships as identifiers of exceedingly granular taxonomic incongruences.

The second solution complements the use of singular, synthetic classifications or phylogenies (see Hinchcliff *et al.* 2015). This solution does not favor one “best” taxonomic inference scheme over another. Instead, the goal is to be logically explicit about the similarities and differences between taxonomies, that is, to resolve the evolution of human taxonomic inference making more formally and precisely than is feasible with names and nomenclatural relationships alone. In the terminology of computer science, an individual taxonomy can be modeled as an ontology (Franz and Thau 2010; Midford *et al.* 2013), and therefore the process of reconciling taxonomic meanings across multiple classifications is a special case of *ontology matching* (Euzenat and Shvaiko 2013; Leonelli 2013). The objective of *aligning* multiple nonidentical taxonomies is in line with broad trends in the data-driven sciences toward semantically tracking the identity and provenance of scientific information, with wide-ranging benefits for data and workflow management (Cheney *et al.* 2007; Zhao *et al.* 2009). Moreover, because ontology interrelationships can be described formally, inferences of multi-taxonomy alignments can be enhanced through application of logic representation and reasoning methods (van Harmelen *et al.* 2008; Bonatti *et al.* 2011).

Here we demonstrate that multi-taxonomy alignments are tractable for two incongruent primate classifications—that is Groves (1993) and Groves (2005), corresponding to the second and third MSW editions— through a combined approach of taxonomic concept representation and logic reasoning. Due to the novelty of our approach, we focus initially on its technical execution for this particular use case. However, the benefits of leveraging computational logic toward the integration of classifications and phylogenies are potentially relevant to any comparative analysis in which taxonomy is an evolving “variable.” We turn to issues of broader implementation and significance in the Discussion.

Our approach has three components. The first is to individuate taxonomic name usages through the name sec. author convention introduced by Berendsohn (1995). The sec. (*secundum*) stands for *according to*, and facilitates the use of distinct *taxonomic concept labels* in which the same taxonomic name can participate(s). For instance, the labels Cebidae sec. Groves (1993) versus Cebidae sec. Groves (2005) are managed as nonidentical symbols and may therefore symbolize noncongruent meanings. Properly individuated concepts can be assembled into entire concept hierarchies (ontologies) via parent/child (*is_a*) relationships (Thau and Ludäscher 2007). This way each separately published perspective—Primates sec. Groves (1993) versus Primates sec. Groves (2005)—can be represented from the ordinal to the species level.

The second component involves providing an initial, limited set of Region Connection Calculus (RCC-5) articulations (Randell *et al.* 1992) that express the extent of taxonomic equivalence among concepts pertaining to distinct hierarchies. The available RCC-5 articulations are: congruence (==), proper inclusion (>), inverse proper inclusion (<), overlap (><), and exclusion (| or !) (Koperski *et al.* 2000; Franz *et al.* 2008; Franz and Peet 2009; Franz and Cardona-Duque 2013; Weakley 2015). Such input articulations are provided by humans with expertise in the corresponding groups, and reflect their understanding of taxonomic meaning relationships in light of the available evidence (e.g., subsumed concepts, homonymy/synonymy, circumscriptions of the phenotype and genotype, distributional data, etc.). For instance, the articulation Cebidae sec. Groves (1993) >< Cebidae sec. Groves (2005) recognizes that each concept entails certain congruent subcomponents, yet each also contains additional subcomponents that are unique to it. Uncertainty can be expressed with RCC-5 in the form of multiple, disjoint articulations (e.g., concept 1 == *or* > concept 2).

Jointly the two input taxonomies (T_1_, T_2_), articulations (A), and additional constraints (C) that apply to most taxonomic hierarchies constitute a set of input constraints (Thau and Ludäscher 2007). The logical consistency of these constraints—that is, which *possible world* scenarios exist that satisfy them—can be assessed through a logic reasoning process. This process, in turn, can produce additional articulations that are logically implied by the input. Hence the third component of our approach aims to produce a logically consistent, exhaustive, and maximally expressive alignment of the input taxonomies. Such an alignment, also called merge taxonomy, in effect constitutes a map of logical congruence relationships that span across the evolving taxonomies.

Multi-taxonomy alignments can inform the integration or separation of biological data initially linked to only one taxonomy, achieving finer degrees of taxonomic resolution than name-based integration methods. The potential and limitations inherent in this approach are illustrated here with an 800-concept input data set, the largest to date for which logic-based taxonomy alignments have been performed (Franz *et al.* 2015, 2016; Jansen and Franz 2015).

The Primates sec. Groves (1993 – MSW2) to Primates sec. Groves (2005 – MSW3) alignment use case (henceforth: Prim-UC) is analyzed with the Euler/X software toolkit (Chen *et al.* 2014a, 2014b, 2015). This open source toolkit can represent and reason over multi-taxonomy constraints using RCC-5 articulations in combination with Answer Set Programming (Brewka *et al.* 2011) and custom reasoners. We first describe the toolkit and basic workflow, including input data formats, workflow interactions, and output products. We then characterize the particular taxonomic input conditions for the Prim-UC, and the pragmatic approach used to articulate the MSW2/MSW3 concepts and achieve consistent alignment outputs. Several partitions of the entire 800-concept data set are made to illustrate different alignment resolution phenomena across taxonomic groups and levels. Because the RCC-5 articulations provide an additional semantic integration layer, the Prim-UC facilitates quantitative assessments of the evolution of name:meaning identity between the input taxonomies (Franz *et al.* 2016). Such analyses are presented for the partitioned merges and the entire alignment. The results have potentially wide-ranging implications for managing taxonomic concepts in the phylogenetic, comparative, and biodiversity information domains. In the Discussion, we suggest pathways to incorporate concept-level representations and alignments to better integrate the evolving stages of synthetic biodiversity and tree of life data platforms.

## Methods

### Reasoning Toolkit and Workflow

**FIGURE 1:**
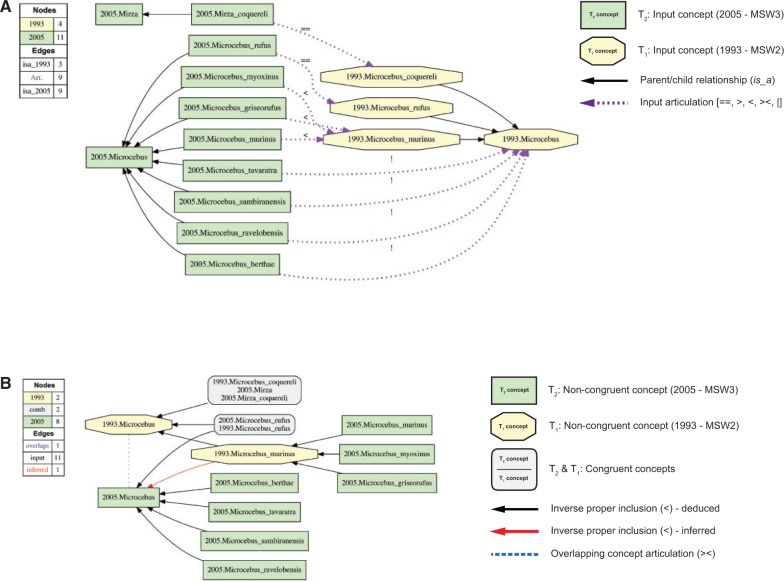
Illustration of the RCC-5 multi-taxonomy alignment approach. In all visualizations (Figs. 1, 3–7, Supplementary Figs. S3 1–13 available on Dryad), the input and aligned, noncongruent concepts sec. Groves (2005) are illustrated as green rectangles (T_2_). Input and aligned, noncongruent concepts sec. Groves (1993) are shown as yellow octagons (T_1_). Congruent sets of aligned concepts are rendered in gray rectangles with rounded corners. A) Visualization of input constraints T_2_ (*Microcebus/Mirza* sec. Groves (2005)), T_1_ (*Microcebus* sec. Groves 1993), and articulations (A) as provided by the user. Each taxonomic concept hierarchy is separately assembled via parent/child (*is_a*) relationships. The three concepts (*Microcebus griseorufus, Microcebus murinus, Microcebus myoxinus*) sec. Groves (2005) are each properly included (<) in *Microcebus murinus* sec. Groves (1993), based on synonymy information shown in Figure 2. Four additional species-level concepts sec. Groves (2005) are articulated as exclusive (|) of *Microcebus* sec. Groves (1993), because they are based on phenotypic material for which there was no equivalent in the earlier (1993) edition of MSW2 (Zimmermann *et al.* 1997; Rasoloarison *et al.* 2000). The legend indicates the number of nodes and edges for each input taxonomy, and the number of user-provided input articulations. B) Visualization (reduced containment graph) of the logically consistent alignment corresponding to the input constraints of (A), showing reasoner-inferred non-/congruent concepts and articulations (see legend). One of two consistent alignments (possible worlds) is shown. The monotypic genus-level concept *Mirza* sec. Groves (2005) *and* its child *Mirza coquereli* sec. Groves (2005) are taxonomically congruent under the coverage constraint where parent concepts are circumscribed by the union of their children (Thau and Ludäscher 2007). Each is therefore also congruent with *Microcebus coquereli* sec. Groves (1993). The two genus-level concepts *Microcebus* sec. Groves (2005) and *Microcebus* sec. Groves (1993) are overlapping; they share two congruent subordinate concepts in the alignment, while also including reciprocally unique children. The reasoner infers 44 logically implied articulations to constitute the set of MIRs, given an input of nine articulations (see also Supplementary Materials S1 and S2 available on Dryad).

The Euler/X software toolkit consists of a set of programming scripts, multiple logic reasoners, and a tree graph visualization system (Thau *et al.* 2009; Chen *et al.* 2014a, 2014b, 2015; Dang *et al.* 2015; Franz *et al.* 2015, 2016; Jansen and Franz 2015). Given an initial set of input constraints (T_1_, T_2_, A, C), the toolkit delivers the following products (Fig. 1). (i) Visualization of each input taxonomy in the format of an *is_a* hierarchy. (ii) Visualization of two input taxonomies and of the set of user-provided articulations (Fig. 1A). (iii) Analysis of logical consistency—if the input is not consistent then no alignment is obtained. (iv) Logic-based diagnosis and removal of constraint inconsistencies (over-specification), requiring resubmission of modified input constraints and return to (iii). (v) Inference and presentation of one or more consistent alignments (possible worlds)—including additional, logically implied articulations—in two data formats: (a) as the set of Maximally Informative Relations (MIRs), interpretable by humans and computers); and (b) as alignment visualizations, and primarily to aid human comprehension (Fig. 1B). (vi) Provision of aggregate views for multiple alignments (constraint under-specification), and decision tree-based reduction of ambiguity in the set of input articulations, leading to more expressive alignments.

A detailed account of the workflow interaction facilitated by the toolkit is provided in Franz *et al.* (2015). Here we focus on reporting consistent, well-specified, and methodologically uniform results for the Prim-UC. We provide all input data files, toolkit commands, and output files (see Supplementary Materials available on Dryad at http://dx.doi.org/10.5061/dryad.6jg71). We have prepared the entire Prim-UC as an experiment for reproduction at http://recomputation.org/ (Gent, 2013). This approach ensures full transparency and permanent accessibility of our data, tools, analyses, and products. To avoid overburdening the narrative with detail on initial, variously over- or under-specified input configurations and necessary repair actions, we opt to address these issues elsewhere.

### Taxonomic Characteristics of the Primate Use Case

**FIGURE 2:**
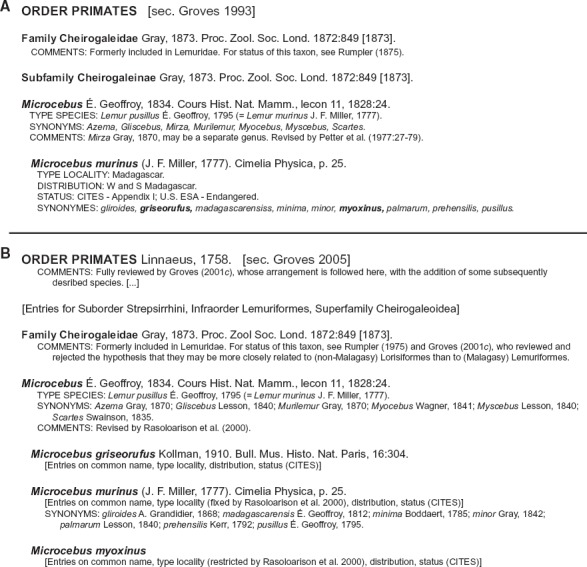
Representation of taxonomic concept entries in the second and third editions of the *Mammal Species of the World* series (Wilson and Reeder 1993, 2005). A) Concept sequence from Primates to *Microcebus* (in part) sec. Groves (1993) (MSW2: 243). B) Concept sequence from Primates to *Microcebus* (in part) sec. Groves (2005) (MSW3: 111–113) (several intermediately ranked concepts omitted). Nine synonymous names are listed under *Microcebus murinus* sec. Groves (1993). Of these, seven are congruently listed under *Microcebus murinus* sec. Groves (2005). However, phenotypes referred to as *Microcebus griseorufus* and *Microcebus myoxinus* (listed in bold italics in A) are treated differentially across treatments, acquiring separate species-level concept status from *Microcebus murinus* sec. Groves (2005) in the MSW3 edition. Accordingly, we can specify the articulations (in abbreviated annotation): (i) 2005.Microcebus_griseorufus < 1993.Microcebus_murinus; (ii) 2005.Microcebus_murinus < 1993.Microcebus_murinus; and (iii) 2005.Microcebus_myoxinus < 1993.Microcebus_murinus. See also Figure 1.

The Prim-UC is based on two input taxonomies. The MSW3 edition directly succeeds the MSW2 edition, with the former intentionally referring to the latter where indicated. These taxonomies are highly consistent in their global scope and structure of presentation (Wilson and Reeder 1993, 2005). Both were published by the same author, Professor Colin Groves (1993), 2005). Moreover, the majority of changes across editions are rooted in an in-between reclassification, also authored by Groves 2001a, 2001b). Although the MSW3 edition follows a more information-rich format, both editions provide the following information (where applicable) for each taxonomic concept entry (Fig. 2): valid scientific name; author, year, and citation of the name priority-carrying publication; common name (MSW3 only); type taxon (name) and complete citation; type locality; conservation status (CITES, IUCN); synonyms; and taxonomic comments. The lists of synonymous names are intended to be comprehensive. Additional comments are meant to clarify taxonomic perspectives and relationships to other treatments that are either in accordance with the respective edition or were not adopted for certain reasons.

In spite of the above similarities, the two input taxonomies of the Prim-UC vary significantly in classificatory perspective (Table 1). The taxonomic differences can be divided into several categories. These include: (i) novel recognition of multiple higher-level ranks for primates in MSW3 (suborder, infraorder, parvorder, superfamily); (ii) changes in the mid- to lower-level concept arrangements (family, subfamily, genus); and (iii) additions of primate species-level concepts in MSW3, due to either (a) an adherence to more narrowly circumscribed concepts, or (b) the accommodation in MSW3 (2005) of primate phenotypes newly discovered and described *after* MSW2 went to press, that is, for which there are no taxonomic equivalents in the earlier (1993) perspective. In all, MSW2 recognizes 317 taxonomic concepts, of which 233 correspond to the species level, whereas MSW3 accounts for 483 taxonomic concepts, with 376 at the species level. This means that 86% (143/166) of the differential in the number of primate taxonomic concepts between MSW2 and MSW3 is grounded in the later taxonomy’s recognition of more species-level concepts than the earlier edition.

**TABLE 1:** Numbers of taxonomic concepts in the Prim-UC listed per rank, with differentials between the two input taxonomies

Taxonomic rank	sec. Groves (1993)	sec. Groves (2005)	Differential
Order	1	1	—
Suborder	—	2	+2
Infraorder	—	5	+5
Parvorder	—	2	+2
Superfamily	—	4	+4
Family	13	15	2 +
Subfamily	10	9	–1
Genus	60	69	9 + 143
Species	233	376	+
Total	317	483	+166

### Provision of Input Articulations

The input taxonomies for the Prim-UC may be viewed as compendia that are taxonomically comprehensive and authoritative (Patterson 1994; Solari and Baker 2007). However, each concept entry is treated in an abbreviated form (Fig. 2). Information on diagnostic features or synapomorphic characters that would characterize revisionary and phylogenetic publications is typically omitted. We nevertheless regard these taxonomies as sufficiently well specified to generate concept-to-concept articulations (Figs. 1 and 2). In particular, we provide single “hybrid” articulations for concept pairs, without distinguishing between intensional (property-referencing) and ostensive (member-referencing) concept components (see Franz and Peet 2009; Franz and Cardona-Duque 2013; Franz *et al.* 2015).

Input articulations were provided with emphasis on the species level (Figs. 1 and 2). The articulations take into account nomenclatural information, synonymy relationships, lower- and higher-level concept arrangements in each input taxonomy, and additional comments. In many instances, this information was sufficient to provide unambiguous species-level articulations. Where needed, primary literature referenced in Groves (2005) was consulted to resolve articulations (e.g., Groves 2000; Rasoloarison *et al.* 2000; van Roosmalen *et al.* 2000).

We stress that specifications of taxonomic concept articulations are not “objective” (Franz *et al.* 2015). Our alignment approach does not represent taxonomic or phylogenetic meaning relationships directly (Cui 2012), but instead proceeds over an asserted *RCC-5 translation* of such evidence. Different users and representational motives may therefore produce alternative alignments for the same input taxonomies. The reliance on user interpretation is an essential part of the workflow. This is not to say, however, that *any* articulation established between two concepts is equally well based in evidence. For instance, the taxonomic concept label Primates sec. Groves (2005) ostensibly refers to (i) *all* taxonomic concepts subsumed under the Primates sec. Groves (1993), *plus* (ii) additional taxonomic entities discovered and described after the publication of MSW2. This circumstance instantly eliminates articulations such as inverse proper inclusion (<), overlap (><), or exclusion (|) from the set of justifiable relationships. The remaining options, that is Primates sec. Groves (2005) == or > Primates sec. Groves (1993) remain adequate under certain interpretations. Similarly, if all relevant information for two species-level concepts pertaining to the two MSW2/MSW3 taxonomies is congruent, and no data that would suggest taxonomic instabilities between them are available, then it is unsound to indicate any articulation other than congruence (==).

**FIGURE 3:**
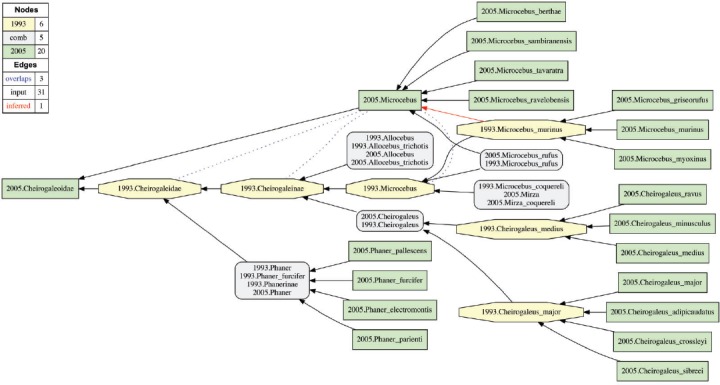
Visualization of the consistent, well-specified Cheirogaleiodae sec. Groves (2005)(T_2_) / Cheirogaleidae sec. Groves (1993)(T_1_) alignment; see also Figures 1 and 2.

When articulating species-level concepts == sec. Groves (2005) to their counterparts sec. Groves (1993), we first determine whether concepts newly recognized at this rank in MSW3 are based, either exclusively or overwhelmingly, on *reassessments* of taxonomic boundaries for phenotypic material and/or variation (“morphospace”; see Pigliucci 2012) *already* recognized at the time of publication of the MSW2 compendium. This is apparently the case for 119 of the 143 newly validated species-level concepts sec. Groves (2005). Examples of such reassessment-contingent species concept additions include *Microcebus griseorufus* Kollmann 1910 sec. Groves (2005) and *Microcebus myoxinus* Peters 1852 sec. Groves (2005). Taxonomic complexities notwithstanding (Rasoloarison *et al.* 2000), the names that participate in the aforementioned taxonomic concept labels were originally anchored by phenotypic material that had been recognized (at least implicitly) in MSW2. We therefore represent these articulations as instances of inverse proper inclusion (<), where union of the more narrowly circumscribed species-level concepts is congruent with the singular, more widely circumscribed concept (Fig. 1). Importantly, the new reassessment-contingent species-level concepts are not considered to expand the circumscriptions of their superordinated parent concepts in the respective taxonomies (Fig. 3).

On the other hand, we identified 24 of the 143 additional species-level concepts sec. Groves (2005) as being based (almost) exclusively on phenotypic material that was newly accessioned and evaluated *after* the publication of MSW2 (see also Reeder *et al.* (2007); Supplementary Materials S4 available on Dryad). Because this material was not deemed assignable to any previously established species-level circumscriptions, the 24 new species-level concepts are not readily articulated to any preexisting entities in MSW2. Examples of such accession-contingent additions include *Microcebus sambiranensis* Rasoloarison, Goodman & Ganzhorn 2000 sec. Groves (2005) and *Microcebus tavaratra* Rasoloarison, Goodman & Ganzhorn 2000 sec. Groves (2005). We therefore represent the corresponding articulations as instances of exclusion (|) (Figs. 1 and 3).

Under this pragmatic approach, additions of species-level concepts that represent new material accessions—that is, with no previously cataloged phenotypic/morphospace equivalents—effectively expand the inclusiveness of the superordinated MSW3 parent concepts in comparison to their MSW2 counterparts. By default, the reasoning approach regards the presence of lower-level incongruences as transitive across more inclusive taxonomic ranks (Franz *et al.* 2015). Thus we obtain a highest-level articulation of Primates sec. Groves (2005) > Primates sec. Groves (1993). This articulation reflects (minimally) the historical sequence of human uncovering of primate phenotype diversity, where material grounding of 24 species-level concepts subsumed under the Primates sec. Groves (2005) was not feasible some 12 years before. The implications of this convention are further considered in the Results and Discussion.

### Input/Output Data Formatting, Alignment Partitions, and Toolkit Commands

All Prim-UC alignments were performed with the open source Euler/X software toolkit (Chen *et al.* 2014a; see also Supplementary Materials available on Dryad), installed on a 4 CPU/8 GB RAM Virtual Machine server (http://euler.asu.edu/). Methods for configuring the input constraints and obtaining output alignments are in accordance with Franz *et al.* (2015, 2016). In particular, we produced several partitions of the entire 800-concept alignment, both to demonstrate partition-specific phenomena and to generate visualizations with taxonomic concept labels that retain legibility.

**FIGURE 4:**
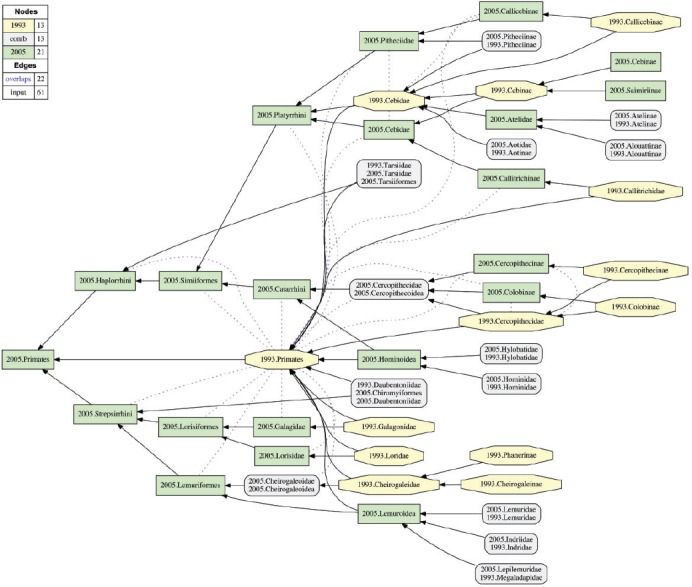
Visualization of the 2005.Primates–Higher-Levels Only alignment (partition 2; see Table 2), with 22 overlapping articulations, of which 16 involve the concept Primates sec. Groves (1993).

**FIGURE 5:**
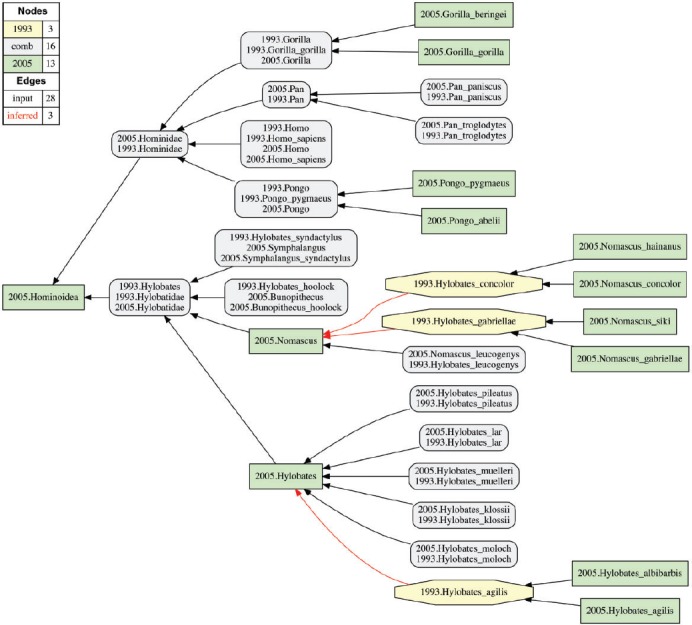
Visualization of the 2005.Hominoidea alignment (partition 6; see Table 2).

We present two sets of six and ten alignment partitions, respectively. The first of these is detailed in Table 2. The six partitions include three alignments of higher-level concepts and their children in the MSW3 taxonomy sec. Groves (2005) with the corresponding MSW2 analogs; namely (i) the suborder-level concept Strepsirrhini sec. Groves (2005) (with an alignment of 124 × 77 input concepts [MSW3 × MSW2]); (ii) the suborder-level concept Haplorrhini sec. Groves (2005), excluding the therein-entailed parvorder-level concept Catarrhini sec. Groves (2005) (169 × 114 concepts); and (iii) the parvorder-level concept Catarrhini sec. Groves (2005) (190 × 125 concepts). Additionally, the set includes: (iv) an alignment of the entire Prim-UC (483 317 concepts); (v) a higher-level subset of that ×comprehensive alignment restricted to the taxonomic rank range of order to subfamily (38 × 24 concepts); and (vi) an alignment of the Hominoidea sec. Groves (2005) and MSW2 analogs (32 × 23 concepts). We show the alignment visualizations either in the main text (Figs. 4 and 5) or in the Supplementary Materials S3 available on Dryad (Supplementary Figs. S3 1–3), with the exception of the entire Prim-UC alignment which is not displayed due to visualization constraints (though see Dang *et al.* 2015).

**TABLE 2:** Alignment partitions for the Prim-UC, showing the highest-level concept for each input taxonomy and number of entailed taxonomic concepts

Partition	sec. Groves (2005)–MSW3	Concepts	sec. Groves (1993)–MSW2	Concepts	Figure
1	Primates	483	Primates	317	—
2	Primates–HLO*	38	Primates*	24	4
3	Strepsirrhini	124	Cheirogaleidae, Lemuridae, Megaladapidae, Indridae,	77	S3–1
			Daubentoniidae, Loridae, Galagonidae		
4	Haplorrhini**	169	Tarsiidae, Callitrichidae, Cebidae	114	S3–2
5	Catarrhini	190	Cercopithecidae, Hylobatidae, Hominidae	125	S3–3
6	Hominoidea	32	Hylobatidae, Hominidae	23	5

*Notes*: Ordered in accordance with taxonomic position and inclusiveness (Fig. 4). *HLO Higher Levels Only. The range of taxonomic ranks is limited to ordinal to subfamiliar level. **Excluding Catarrhini sec. Groves (2005).

The second set of Prim-UC partitions entails ten alignments based on the following mid-level concepts sec. Groves (2005) and their MSW2 counterparts: Cheirogaleoidea (see also Fig. 3), Lemuroidea, Lorisiformes, Chiromyiformes, Tarsiiformes, Platyrrhini (excluding Callitrichinae), Callitrichinae, Cercopithecinae, Colobinae, and Hominoidea (repeated). The corresponding alignment visualizations are provided in the Supplementary Materials S3 available on Dryad (Supplementary Figs. S3 4–13).

We use the conventions of Franz *et al.* (2015, 2016) to configure the Prim-UC input data files (Supplementary Materials S1 available on Dryad), output sets of MIRs;Supplementary Materials S2 available on Dryad), and input/output alignment visualizations. The later MSW3 taxonomy sec. Groves (2005) is consistently represented as T_2_, whereas the earlier MSW2 taxonomy sec. Groves (1993) is labeled as T_1_. This T_2_–T_1_ sequence of annotation is used in all input articulations and output MIR.

To maximize consistency between the narrative and toolkit input format, an abbreviated annotation is used for the taxonomic concept labels, where (e.g.,) Primates sec. Groves (2005) becomes “2005.Primates” and *Microcebus murinus* sec. Groves (1993) becomes “1993.Microcebus_murinus” (Fig. 1). Thereby all 800 concepts are unambiguously symbolized. We utilize the shorthand format in the Results and Discussion.

Two Euler/X toolkit commands were employed to generate the input and output alignments and visualizations. The input data files are annotated with the respective command line arguments. The command “show input visualization” was run to visualize the alignment input (Fig. 1A). The command “polynomial encoding / show possible worlds / reduced containment graph” was used to obtain consistent alignments products, including the sets of MIR (.csv format) and GraphViz-rendered visualizations (.pdf format). The “reduced containment graph” option shows overlapping articulations among input concepts as blue dashed lines in the output visualizations (Fig. 1B) (for further detail see Chen *et al.* 2014a). The ratio of the number of input articulations and output MIR is provided as a measure of information newly made explicit through reasoning (Franz *et al.* 2015). The original toolkit visualizations were minimally edited with the OmniGraffle illustration software (http://www.omnigroup.com/) to obtain consistent spatial renderings of concept groups.

The alignments were inferred with the toolkit’s Answer Set Programming reasoners, with the exception of the entire 800-concept alignment, which was analyzed with a custom-generated RCC-reasoner (Bowers, personal communication). All input files, toolkit scripts, software dependencies, and run commands for the Prim-UC have been submitted for open, permanent access and identical reproduction at http://recomputation.org/ (see Supplementary Materials S5).

### Analyses of Name:Meaning Relations

The output MIR (Supplementary Materials S2 available on Dryad) were variously sorted and compared to perform simple, quantitative name:meaning relationship analyses for the Prim-UC (Geoffroy and Berendsohn 2003; Franz *et al.* 2008, 2016). In particular, for each partition we recorded the number of MIR representing each of the RCC-5 articulations (Table 3). The quotient of (i) the number of congruent articulations (==) in an alignment and (ii) the number of input concepts in the concept-poorer taxonomy (T_1_ in all partitions) provides an approximation of the degree of relative congruence for each partition (Table 5). If the ratio approaches 1:1 then relative congruence is high, possibly despite differences in name usage and taxonomic resolution.

**TABLE 3:** Summary of input concepts, articulations, reasoner-inferred MIRs, and information expression ratio (inferred MIR/input articulations) for the six Prim-UC partitions, with numbers of specific RCC-5 articulations

Partition	sec. Groves (2005)	Concepts	Articulations	MIR	Expression	==	>	<	><	|
1	Primates	800	402	153,111	380.9× 22.8	283	2053	1649	49	149,077
2	Primates–HLO*	62	40	912	× 101.6	13	110	27	24	738
3	Strepsirrhini	201	94	9548	128.4 ×	74	307	246	5	8916
4	Haplorrhini**	283	150	19,266	151.3 ×	98	621	423	5	18,119
5	Catarrhini	315	157	23,750	32.0 ×	111	558	549	11	22,521
6	Hominoidea	55	23	736	×	24	54	63	0	595

*Notes*: See also Table 2. *HLO = Higher Levels Only. The range of taxonomic ranks is limited to ordinal to subfamiliar level. **Excluding Catarrhini sec. Groves (2005).

Focusing on the entire 800-concept alignment, we furthermore resolve name:meaning relationships of MSW3/MSW2 concept pairings by shared taxonomic rank (for MSW2 ranks only; see Table 1), based on the following categories: (i) taxonomic congruence, same name(s) (symbolized as == : =); (ii) taxonomic congruence, different names (== : ≠); (iii) taxonomic proper inclusion, same name(s) (> =:); (iv) taxonomic inverse proper inclusion, same name(s) = (< : =); and (v) taxonomic overlap, same names(s) (>< : =). “Same name(s)” in the present context means: identical strings as represented in the input data files. Because both input taxonomies are Code-compliant and no homonyms are involved, there are no instances of identically named MSW3/MSW2 concept pairings that are taxonomically exclusive of each other (|: =).

We consider the (== : =) category (i) to represent reliable names, whereas the remaining above categories (ii—v) entail concept pairings with unreliable names; that is, either identical names (N_2_ *N*_1_) symbolize noncongruent concepts (C_2_ [>, <, ><] C=_1_), or congruent concepts (C_2_ ==*C*_1_) are symbolized with nonidentical names (N_2_ =*N*_1_). A reliability ratio for MSW3/MSW2 names is calculated for each of the six main partitions as the quotient of reliable and unreliable names, adjusting the lower value to 1 (Table 5). Thus a 1:1 reliability ratio would indicate that half of the name:meaning relations in the five-category set are of the (== : =) type for a given alignment.

## Results

We focus the Results and Discussion on the outcomes and implications of our alignment approach, as opposed to detailed assessments of the underlying taxonomic perspectives. In particular, we refer to external publications for insights into alternative mammalian species concepts whose application affects the Prim-UC alignments (see, e.g., Groves 2001a, 2001b, 2012, 2013; Baker and Bradley 2006; Asher and Helgen 2010; Frankham *et al.* 2012; Gippoliti and Groves 2012; Heller *et al.* 2013; Zachos *et al.* 2013; Zachos and Lovari 2013; Cotterill *et al.* 2014).

### Characterization of Alignments and Causes for Taxonomic Incongruence

The sets of six and ten input partitions yield a single, well-specified alignment in each case (Figs. 4 and 5 and Supplementary Figs. S3 1–13 available on Dryad). The consistent taxonomic scope, information organization, and recursive reference relation among the input taxonomies contribute to the well-resolved outcomes. However, the high degree of resolution is paired with frequent and heterogeneous occurrences of taxonomic incongruence, as is reflected in the spatial distribution of green rectangles (concepts unique to Groves 2005) and yellow octagons (concepts unique to Groves 1993) in the alignment visualizations.

Much of the taxonomic incongruence is rooted in relatively higher numbers of species-level concepts in MSW3 (Table 1). Whereas 119/143 such instances (~83%) are reassessment-contingent (i.e., primarily due to narrower species concept delimitations applied in MSW3), the remaining 24 species-level concept additions are grounded in newly accessioned specimen material that was not available for the MSW2 compendium and cannot be subsumed under the earlier species-level concepts (see also Supplementary Materials S4 available on Dryad). Narrower taxonomic resolution in MSW3 is also evident at the generic level. Examples include monotypic concepts such as *Mirza* sec. Groves (2005), *Prolemur* sec. Groves (2005), *Pseudopotto* sec. Groves (2005), *Oreonax* sec. Groves, *Symphalangus* sec. Groves (2005), and *Bunopithecus* sec. Groves (2005). Each of these is congruent with species-level concepts already recognized in Groves (1993), yet therein symbolized by other genus-level name/epithet combinations. Interestingly, the balance of the MSW3/MSW2 narrow/wide concept pattern is not fully one-sided. Groves (1993) recognizes four species-level concepts that jointly correspond to the two concepts *Aotus azarae* sec. Groves (2005) and *Aotus lemurinus* sec. Groves (2005) (Supplementary Figs. S3– 2 and S3–9 available on Dryad). The MSW3-recognized synonyms *Aotus infulatus* (Kuhl) and *Aotus brumbacki* Hershkovitz are considered valid species-level names in Groves (1993).

The high-level view of the Prim-UC alignment (Fig. 4) reveals that certain instances of lower-level incongruence propagate to superordinated ranks. For instance, the overlapping articulation 2005.Microbus >< 1993.Microcebus “cascades up” to the family rank in the form of 2005.Microcebus >< 1993.Cheirogaleidae (Fig. 3). The species-level provenance for this articulation is also depicted in Figure 1. In other instances where only reassessment-contingent differences are present at lower levels, these differences can integrate up to congruent superordinated concepts. Examples of the latter are as follows: 2005.Hylobatidae == 2005.Hylobatidae and 2005.Hominidae == 1993.Hominidae (Fig. 5).

Groves (2005) introduces three additional, higher-level elements causing incongruence in relation to Groves (1993) (Fig. 4). The first of these are added partitions above the family level: either (i) MSW3-endorsed superfamily-level concepts such as 2005.Lemuroidea and 2005.Hominoidea aggregate multiple reciprocally congruent family-level concepts, or (ii) monotypic concepts like 2005.Chiromyiformes and 2005.Tarsiiformes are taxonomically congruent with their immediate respective children 2005.Daubentoniidae and 2005.Tarsiidae. The second kind of taxonomic incongruence is related to the subfamily level. In particular, Groves (1993) recognizes two subfamily-level concepts 1993.Cheirogaleinae and 1993.Phanerinae whose names have no valid or synonymous status in Groves (2005)—there are simply no analogs in MSW3. Conversely, Groves (2005) introduces the concept 2005.Saimiriinae for which there is no nomenclatural or taxonomic counterpart in Groves (1993).

**FIGURE 6:**
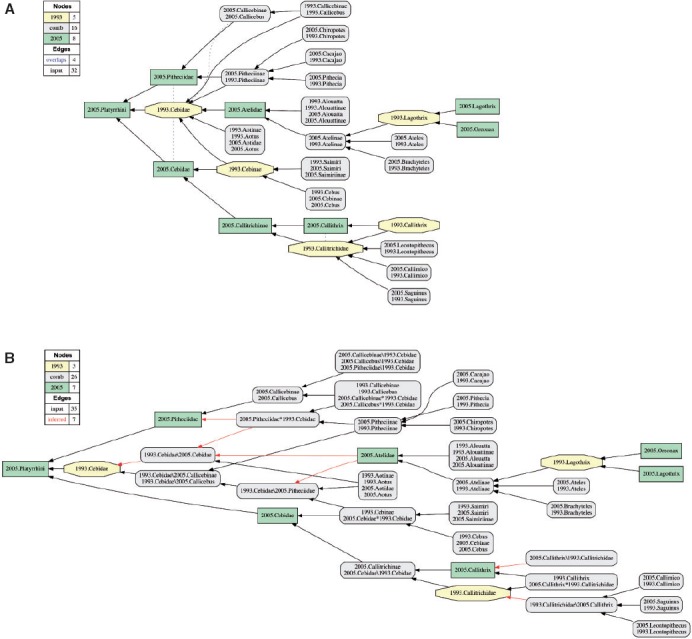
Visualization of the parvorder- to genus-level alignment corresponding to the 2005.Platyrrhini and 1993.Callitrichidae/1993.Cebidae (see also Supplementary Fig. S3–2 available on Dryad). A) Reduced containment graph visualization, with overlap shown. B) Combined concept visualization (see Franz *et al.* 2015), using the C_1_\C_2_, C_2_\C_1_, (where “\” = *not*), C_1_^∗^C_2_ (where “*” *and*) annotation to identify Euler subregions that result from input concept overlap. Recognizing such newly inferred Euler subregions in the=alignment increases the number of inferred articulations (red arrows).

The third kind for higher-level incongruence is due to more profound taxonomic differences. Most striking in this regards are the alternative perspectives on taxonomic concepts that Groves (1993) assigns to the 1993.Cebidae (Figs. 4 and 6). This widely circumscribed family-level concept has overlapping articulations with the concepts 2005.Cebidae and 2005.Pitheciidae, and additionally properly includes the concepts 2005.Aotidae and 2005.Atelidae.

**FIGURE 7:**
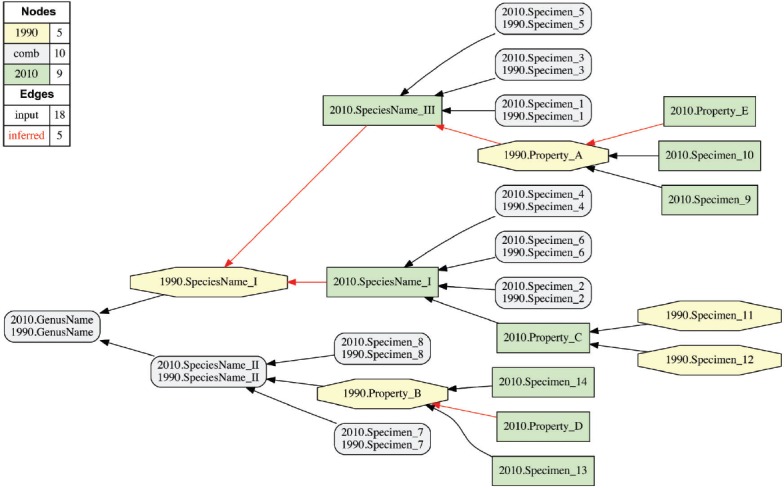
Hypothetical alignment (1/2 possible worlds shown) whose input entails two incongruent sets of taxonomic concepts, character concepts, and specimen-to-taxonomic concept or specimen-to-character concept assignments. The earlier (1990) and later (2010) treatments involve overlapping sets of specimens, where Specimens 1–8 are shared (i.e., re-/examined in each treatment), Specimens 9–10 and 13–14 are only included in the 2010 treatment, and Specimens 11–12 are exclusively observed in the 1990 treatment. The five species-level taxonomic concepts respectively include 2–5 Specimens and one Property (A–E). Specimens are either directly assigned to parent concepts (as their *is_a* children), or are *identified to* properties (which are represented as concepts in themselves) via (>, <) articulations. Only two concept-to-concept articulations are provided: (i) 2010.SpeciesName_II 1990.SpeciesName_II and (ii) 2010.GenusName == 1990.GenusName. The reasoning process yields an alignment with unambiguous articulations among higher-level concepts and their==properties that are logically grounded in the specimen-level articulations.

Overall, the higher-level alignment contains 22 overlapping articulations (Fig. 4), of which only three are redundantly depicted in the 2005.Haplorrhini alignment (Supplementary Fig. S3–2 available on Dryad) and two are represented in the 2005.Catarrhini merge (Supplementary Fig. S3–3 available on Dryad). The remaining 16 overlapping articulations involve the root-level concept 1993.Primates. The relative congruence is lowest for this alignment with 54.2%, whereas the other focal alignments vary between 86% and 100% for this measure (Table 5).

Further analysis shows that certain mid- to lower-level alignments are more dynamic than others. For instance, relative to the Strepsirrhini sec. Groves (2005), we observe more changes in the subsumed 2005.Cheirogaleoidea alignment (Fig. 3 and Supplementary Fig. S3–4 available on Dryad), with a ratio of 6:25 congruent:unique concept regions, than in the 2005.Lemuroidea alignment, with a 22:31 ratio (Supplementary Fig. S3–5 available on Dryad). Similarly, the 2005.Colobinae alignment (Supplementary Fig. S3–12 available on Dryad), with a 29:50 ratio, is indicative of more incongruence than the 2005.Hominoidea alignment (Fig. 5 and Supplementary Fig. S3–13 available on Dryad), with a 16:16 ratio. These differences are partly due to unequal numbers of acquisition-contingent species-level concept additions—four in the 2005.Cheirogaleoidea versus zero in the 2005.Lemuroidea, and two in the 2005.Colobinae versus zero in the 2005.Hominoidea. They also appear to reflect differences in the quantity and quality of evidence driving taxonomic and phylogenetic inferences across the 1993–2005 interval, in particular with regards to certain Malagasy and Neotropical primate lineages.

### Information Expression and Input Sufficiency

The Prim-UC partitions illuminate the relationship between the number of sufficient input articulations and the number of articulations “newly expressed” in the MIRs as an outcome of the reasoning process (Franz *et al.* 2015). This relationship is contingent on globally applied reasoning constraints, which include: nonemptiness, sibling disjointness, and coverage, that is, a parent concept is completely circumscribed by the union of its children (Thau and Ludäscher 2007). Algorithmically, the number of MIR for an alignment is the product of the number of concepts provided in each input taxonomy (Chen 2014).

We speak of information expression achieved through the reasoning, as opposed to information gain, because the full set of MIR for an alignment is logically implied by the input constraints. Strictly speaking, no new information is gained (see also Dececchi *et al.* 2015). From the user perspective, however, the number of explicitly specified MIR in the output exceeds that of the input articulations by one or more orders of magnitude (Table 3). No Prim-UC partition has disjoint (ambiguous) input or output articulations, and consequently a single alignment is obtained in each case.

The ratio of input articulations (A) to input concepts (T_1_, T_2_) is approximately 1:2 across all alignments. The highest ratio (40:62) is present in the higher-level 2005. Primates alignment, and the lowest ratio (23:55) is observed in the 2005.Hominoidea alignment. Whereas the numbers of sufficient input articulations per alignment range from 23 to 402 articulations (factor of ∼17*.*5×), the corresponding numbers of output MIR range from 736 to 153,111 (factor of 208×). Accordingly, the rate of information expression∼ varies from 22.8× in the most thoroughly constrained higher-level 2005.Primates alignment, to 380.9× in the entire 800-concept alignment.

Due to global application of the coverage constraint, the alignment of higher-level (parent) concepts is “driven” by input articulations between their respective lower-level (child) concepts (Fig. 1A). For instance, the 2005.Cheirogaleoidae alignment (Fig. 3) entails 21 species-level concepts sec. Groves (2005). Of these, three are congruent with same-ranked concepts sec. Groves (1993); 14 represent reassessment-contingent, narrower concepts that jointly align with only four species-level concepts sec. Groves (1993); and four are acquisition-contingent additions. The 21 corresponding MSW3/MSW2 species-level articulations, plus one additional articulation 2005.Cheirogaleoidae > 1993.Cheirogaleidae, are sufficient to yield the single, consistent alignment. The input is sufficiently well specified in spite of 11 unarticulated, intermediate-level concepts—nine at the genus level, and two at the subfamily level. Indeed, providing unambiguous species-level articulations, and then adding one or few highest-level(s) articulations, was sufficient to yield well-specified alignments for all partitions. For instance, the 402 input articulations specified for the 800-concept alignment are only 26 more than needed to articulate each of the 376 species-level concepts sec. Groves (2005) at least once. Unambiguous MIR articulations for approximately 80/483 concepts sec. Groves (2005) (16.6%) were derived in the absence of any directly referencing input articulations, that is, solely on the basis of logically propagating the sufficient signal from input articulations that involve other concepts.

### Analyses of Name : Meaning Relations

Concept-level resolution of taxonomic congruence in the Prim-UC demonstrates frequent name:meaning dissociation between the two MSW editions. Some measure of dissociation necessarily follows from the 143 species-level concept increase in MSW3, which typically means that an inverse proper inclusion:same name(s) (< : =) relationship is created for homonymous 2005/1993 concepts. An example of this relationship is the articulation 2005.Microcebus_murinus < 1993.Microcebus_murinus (Figs. 1–3). In total, 55 such instances occur in the Prim-UC (Table 4).

**TABLE 4:** Analysis of taxonomic name:meaning relations for the entire Prim-UC alignment (800 input concepts), grounded in the MIRs (Table 3)

Rank	sec. Groves (2005)	sec. Groves (1993)	== : =	== : ≠	> : =	< : =	>< : =	Totals
Species	376	233	151	17	1	55	0	224
Genus	69	60	44	0	7	6	2	59
Subfamily	9	10	3	0	3	1	0	7
Family	15	13	5	2	1	0	1	9
Order	1	1	0	0	1	0	0	1
Totals	470	317	203	19	13	62	3	300

*Notes*: Relations are categorized by taxonomic rank (for shared MSW2/MSW3 ranks only), and emphasize concept pairs with the same name (=) and/or congruent meanings. Legend: == : =→ taxonomic congruence, same name(s);== : ≠→ taxonomic congruence, different names; > : =→ taxonomic proper inclusion, same name(s); < : =→ taxonomic inverse proper inclusion, same name(s); >< : =→ taxonomic overlap, same names(s).

The entire 800-concept alignment contains 203 reliable names (Tables 4 and 5), herein understood as 2005/1993 concept pairings that are taxonomically congruent (C_2_ ==C_1_) and whose taxonomic names are identical (N_2_ =N_1_). Of these, 151 are species-level names (compared with totals of 376/233 MSW3/MSW2 species-level concepts) and 44 are genus-level names (with totals of 69/60 genus-level concepts). Only eight reliable names are operative above the genus level, in spite of 24 “opportunities” for such name:meaning relations to occur at the subfamily, family, and order levels (Table 4). Unreliable names are mainly of the taxonomic congruence:different names (== : ≠) type (19 articulations) or the aforementioned >==: type (62 articulations). Overlap (><) across identically ranked concepts with the same name(s) is rare; occurring twice at the genus level (*Microcebus* and *Pygathrix* sec. Groves (2005)/1993; Fig. 3 and Supplementary Fig. S3–12 available on Dryad), and once at the family level (Cebidae sec. sec. Groves 2005/1993; Fig. 4). Roughly 3/4 of the unreliable names occur at the species level (224/300 same-ranked concept pairs).

Examination of the reliability ratio, herein taken as the ratio of correlated (== : =) versus dissociated (== : ≠, *not* == : =) name:meaning relations across the two MSW editions, reveals a 2.1:1 reliable:unreliable names relation for the entire Prim-UC (Table 5). Among smaller partitions, the ratio is least favorable for high-level names (partition 2–1:1.5), and most favorable for names used in the 2005.Haplorrhini alignment (partition 4–1.8:1). Even for the 2005.Hominoidea alignment (partition 6), which contains only reassessment-contingent species-level concept additions in relation to its MSW2 predecessor, the reliability ratio is not higher than 1:1. Among the 28 articulations that express either taxonomic congruence (==) or nomenclatural sameness (=), only 14 are of the combined (and ideal) == : = type.

**TABLE 5:** Analysis of taxonomic congruence and name reliability for six Prim-UC partitions (Table 2)

Partition	sec. Groves (2005)	T_1_ concepts	Actual == articulations	Relative congruence (%)	Reliable names	Unreliable names	Reliability ratio
1	Primates	317	283	89.3	203	97	2.1 : 1
2	Primates–HLO*	24	13	54.2	8	12	1 : 1.5
3	Strepsirrhini	77	74	96.1	45	49	1 : 1.1
4	Haplorrhini**	114	98	86.0	79	45	1.8 : 1
5	Catarrhini	125	111	88.8	79	63	1.3 : 1
6	Hominoidea	23	24	100	14	14	1 : 1

*Notes*: Relative congruence is understood as the quotient of the number of congruent concepts and number of concepts in the concept-poorer taxonomy (T_1_; sec. Groves 1993). The quotient may be greater than 100% if the concept-richer taxonomy has “redundant” concepts (i.e., multiple concepts with superseding ranks that are taxonomically congruent; see Gregg 1954). Reliable names are of the : type in Table 4. Unreliable names are of the [== : ≠, > : =, < : =, >< : =] types in Table 4. The reliable : unreliable ratio is adjusted to 1 for the smaller value. *HLO = Higher Levels Only. The range of taxonomic ranks is limited to ordinal to subfamiliar level.

**Excluding Catarrhini sec. Groves (2005).

## Discussion

In the Discussion, we try to balance insights related directly to the Prim-UC with broader questions regarding the significance of our approach and its applicability to other use cases. Due to the complexity of systematic data, we cannot address every conceivable use case scenario. At best, we have developed a logic-based solution to resolving name:meaning conflicts and ambiguities that affect virtually every analysis where primary data are identified to multiple, incongruent source classifications and/or phylogenetic trees (Franz *et al.* 2015). However, the need for such resolution will depend on the inference demands of specific analyses, and the possibility for application will depend on the properties of the input classifications and users’ preparedness to achieve expressive alignments. Hence we discuss the strengths and limitations of our approach as they are apparent now, but must leave certain valid questions open until answers are more thoroughly explored.

After reviewing the outcomes of the Prim-UC, we focus in particular on how adoption of the RCC-5 alignment approach might refine services provided by open-ended biodiversity and phylogeny data assembly platforms that are becoming increasingly central to systematics in the broad sense.

### Feasibility of Logic-Based Multi-Taxonomy Alignments: Prim-UC and Beyond

The Prim-UC demonstrates that logically consistent alignments of biological taxonomies are feasible, and scalable to at least 800 input concepts. The RCC-5 alignment approach provides both human- and machine-interpretable data outputs, in the form of sets of MIRs (Supplementary Materials S2 available on Dryad) and alignment visualizations (Figs. 4 and 5 and Supplementary Figs. S3 1–13 available on Dryad). The reasoning process yields additional, logically implied articulations in numbers two or more orders of magnitude greater than the input articulations. These products measure taxonomic congruence at more granular levels than possible using just taxonomic names and nomenclatural relationships (homonymy, synonymy) or phyloreferences (Bryant and Cantino 2002; Dubois 2005: Franz 2009). They enable quantitative assessments of name:meaning relations across the aligned taxonomies, which in turn can inform data integration practices for multisourced comparative analyses where names are traditionally used to identify taxonomic content (Franz *et al.* 2008).

How widely applicable is this approach? We believe that the answer to this question depends on specific taxonomic resources and assumptions that make an expressive alignment feasible, or not. The Prim-UC was chosen to exemplify conditions under which the RCC-5 alignment approach can succeed. To this end, we provide thorough documentation for reproduction of our analyses (e.g., Supplementary Materials S5). The input articulations are extracted from the content of the two MSW editions (Fig. 2). They are third-party articulations (Franz and Peet 2009), specified “after the fact,” by humans who were not authors of the source classifications. This circumstance is pragmatic rather than ideal, and differs from other analyses where we have played both the role of primary authors of classifications and of alignment providers (Franz *et al.* 2015, 2016; Jansen and Franz 2015). In either case, one key condition for using this approach is to motivate users to thoroughly compare the content of multiple classifications while using RCC-5 articulations. The comparison will often include classifications or trees that are considered incomplete, provisional, or even erroneous from the user’s perspective. Generally speaking, the approach requires users to adopt an attitude that values the current state of taxonomic knowledge as well as long-term knowledge integration needs.

Our distinction between reassessment- and acquisition-contingent species-level concept additions in the MSW3 edition (Groves 2005) is the most consequential semantic assumption used to generate the Prim-UC alignments. Three related objections may be offered against this distinction. In discussing the merits of each in the following paragraphs, we also characterize the suitability of the RCC-5 alignment approach for other use cases.

First, it may be objected that the reassessment/acquisition distinction is not sound, because taxonomic revisions almost always include unequal sets of specimens. These sets represent mixtures of previously assessed and newly acquired material (Pullan *et al.* 2000; Dikow and Meier 2004). It is commonplace in phylogenetic and revisionary systematics to combine partial resampling of previously analyzed material with newly acquired specimens to produce novel syntheses. Nevertheless, it is semantically meaningful to differentiate (i) whether “new” species-level names are fundamentally derived by reinstating preexisting and synonymous names and associated types as valid, or (ii) whether the discovery of thus far undescribed phenotypes requires the creation of new names and concepts that have no overlap with already published-on species-level entities. The former case can be thought of as recognizing finer subdivisions of previously charted geno-/phenospace. The latter case, in turn, genuinely expands that space in relation to our most recent taxonomies. Thus, although the reassessment/acquisition distinction is not the only form to account for the differential of 143 species-level concepts sec. Groves (2005), it is taxonomically adequate and conducive to particular representation and reasoning needs.

The second objection is directly related to the first, and concerns our global acceptance of the coverage constraint (Thau and Ludäscher 2007). An alternative way to voice this concern is to point out that the Prim-UC articulations among parent-level (above species-level) concepts do not reflect feature-based relations. The objection is related to the notion of representing taxonomic concepts as classes and/or individuals (see Franz and Thau 2010), and more concretely, whether the congruence between parent-level concepts should be based solely on the signal cascading up from their respective children. In the present analysis, this is the case.

Applying the coverage constraint strictly across the full depth of the input taxonomies means that only one acquisition-contingent species-level addition in MSW3 will effectively render the Primates sec. Groves (2005) properly inclusive of (>) Primates sec. Groves (1993) (Fig. 4). However (as one might argue), the inference that somehow the order-level circumscription of 2005.Primates has expanded over that of 1993.Primates—just by virtue of adding one or more newly discovered species-level concepts—is unintuitive in the following sense. We can conceive of a counterfactual situation in which Professor Groves had had access to the analysis and specimen material pertaining to *Microcebus sambiranensis* sec. Rasoloarison *et al.* (2000) *at an earlier time*, that is, when he crafted the MSW2 contribution (Groves 1993). Under that counterfactual scenario, we may ask: had the expert examined this taxonomic entity and its associated material, would he have subsumed it under Primates sec. Groves (1993)? Moreover, would he have assigned the entity to the genus-level concept *Microcebus* sec. Groves (1993), in accordance with inferences made in Rasoloarison *et al.* (2000)?

We sense that the first question can be answered affirmatively. Groves (1993) ought to have recognized that specimens pertaining to *Microcebus sambiranensis* sec. Rasoloarison *et al.* (2000) are to be classified under “primates” (1993.Primates). Quite likely, he would have also considered them to pertain to the “mouse-lemurs” (1993.Microcebus) as circumscribed in Groves (1993). Indeed, he approved of this generic placement in his MSW3 contribution (Fig. 3). So then, under what conditions is it adequate to indicate that 2005.Microcebus_sambinarensis is exclusive of (|) 1993.Primates, and hence 2005.Primates > 1993.Primates? And conversely, when is it justified to indicate that 2005.Microcebus_sambinarensis is included in (<) 1993.Primates, and hence 2005.Primates == 1993.Primates?

The former representation convention (2005.Primates > 1993.Primates, etc.) characterizes the current Prim-UC alignments. The alternative—that is, 2005.Primates == 1993.Primates, etc.—is feasible, but only under an *intensional reading* of articulations between parent-level concepts (Franz *et al.* 2008; Franz and Peet 2009; Franz *et al.* 2015). For instance, we could circumscribe 1993.Primates with the putative synapomorphic trait: “tympanic floor fully ossified, petrosal plate major element, it forms anterior, medial, and posterior walls.” This trait (concept) was inferred to represent one of several “good synapomorphies” for Primates sec. Shoshani *et al.* (1996:114 and 131—character 13, state 3). The character/state interpretation is grounded in earlier treatments by Wible and Martin (1993) or MacPhee (1981). Other inferred traits in Shoshani *et al.* (1996; e.g., characters 24, 94) have congruent referential extensions and similar legacies of recurrent taxonomic application.

The predictive nature of feature-based definitions for higher-level taxonomic concepts is precisely what would allow us to *affirm* the counterfactual question raised above. In particular, we may posit that Groves (1993) endorsed the primate synapomorphies inferred in MacPhee (1981). If we then also recognize that specimens of *Microcebus sambiranensis* sec. Rasoloarison *et al.* (2000) “match” these synapomorphic features (i.e., they are observed as present in them), then 2005.Microcebus_sambinarensis *<* 1993.Primates becomes an appropriate articulation. We could similarly accommodate the other 23 acquisition-contingent species-level concepts of MSW3 under an intensionally circumscribed concept of 1993.Primates. This will yield the ordinal-level articulation 2005.Primates 1993.Primates.

Intensional encodings of articulations can represent congruence among parent-level concepts in spite of incongruent sets of entailed children. Such encodings can be applied locally, or globally. When used, feature-based RCC-5 articulations frequently recover intensional congruence between concept pairs whose children were differentially sampled across treatments. This also means that lower-level incongruences need not cascade up to higher-levels (Fig. 3), but instead can be resolved at the next higher levels where congruent, feature-based articulations occur (Franz *et al.* 2015).

Returning to the second objection, the issue is not that it is logically impossible to relax the global coverage constraint and thus obtain property-centric— and likely more congruent—alignments at higher levels. Such alignments are possible in principle and practice, offering flexibility in semantic representation where desired by the user. We opted not to specify feature-based RCC-5 articulations here because of the specific content of the Prim-UC source classifications. Neither input taxonomy directly provides feature-based circumscriptions of higher-level concepts (Fig. 2). Asserting such information should be the task of an expert speaker with very immediate access to intensional readings of concepts in each classification (Franz and Peet 2009). Indeed, much information *could* be derived from Groves (2001a); for instance, the divergent feature-based diagnoses of *Microcebus* sec. Groves 2001a:68–69) versus *Mirza* sec. Groves 2001a:70–71). We choose to leave this task to others.

The larger points about the applicability of the RCC-5 alignment approach that emerge from this discussion are as follows. (i) The approach is potentially widely applicable to phylogenies and/or classifications that have labeled parent-level concepts, where the latter may also entail feature-based circumscriptions. Different alignment needs can be addressed by providing member- and/or feature-based input articulations. (ii) The users’ abilities and needs to translate the taxonomic signals coming from multiple input sources into RCC-5 articulations are an essential but not yet fully understood constraint of this approach.

The third and last objection might challenge the high degree of resolution in the Prim-UC alignments. One might argue that this resolution exceeds the precision of the information provided in the input classifications. Indeed, our strict reassessment/acquisition distinction has produced 376 unambiguous species-level articulations, leading to unique merge taxonomies for each input partition (Figs. 3–5 and Supplementary Figs. S3 1–13 available on Dryad). This is so despite the fact that many currently recognized primate groups have complex taxonomic histories; for instance *Cheirogaleus* (Groves 2000, 2001a) or *Microcebus* (Tattersall 1982; Groves 2001a). Perhaps no unambiguous articulations should be regarded appropriate in such cases.

In our view, this last objection does not concern feasibility so much as utility. The RCC-5 alignment approach represents uncertainty as the absence of certainty; that is, ambiguity is expressed by providing disjoint input articulations. For instance, if no taxonomic information is available for two concepts labeled 2005.Microcebus_murinus and 1993.Microcebus_murinus (other than: that they are not homonyms), we can still articulate with confidence: 2005.Microcebus_murinus [== or > or < or ><] 1993.Microcebus_murinus. Exclusion (|) is not an acceptable articulation because both concepts are anchored by the same nomenclatural type. The disjoint articulation can be logically processed and yield consistent alignments. If many such articulations are present, we can expect many alignments (Franz *et al.* 2015), and may require new visualization tools to explore these (Dang *et al.* 2015). The approach remains feasible even if the content signal from the input taxonomies is ambiguous. However, in cases of very high ambiguity the resulting RCC-5 alignments may not provide much resolution beyond the degree achieved by nomenclatural relationships. Again, the role of (expert) users is critical because of their unique abilities to contextualize taxonomic sources and thus derive more precise signals from them.

In summary, each of three objections to our Prim-UC alignments is potentially valid. Our input articulations may indeed contain errors and over-specifications, and they are not optimized to represent featured-based circumscriptions of parent-level concepts. But, instead of being fundamental limitations of the RCC-5 alignment approach, these concerns reflect use case- and user-specific constraints and preferences, related to the quality of the input signal and particular representation needs. In each case, the appropriate response to the objections is to propose and scientifically justify alternative, consistent alignments. These issues are the proper domain of taxonomic discourse, and should lead to more a nuanced understanding of taxonomic congruence in this and a wide range of other use cases.

### Concept Alignment-Based Resolution of Taxonomically Annotated Data

Why should systematists use this approach, individually and collectively? To address this question, we outline two scenarios that utilize taxonomic alignments toward integration services in open-ended biodiversity data and phylogenetic information platforms. Additional discussions are developed in Berendsohn (1995), Geoffroy and Berendsohn (2003), Franz (2005), Kennedy *et al.* (2005), Franz *et al.* (2008, 2015, 2016), Laurenne *et al.* (2014), Lepage *et al.* (2014), and Remsen (2016). We note, however, that RCC-5 articulations are not yet explicitly tractable with either the well-established Darwin Core biodiversity data or the NeXML phylogenetic data exchange standards (Vos *et al.* 2012; Wieczorek *et al.* 2012; McTavish *et al.* 2015; Baskauf and Webb 2016).

#### Biodiversity data platforms

Consider using the term “*Microcebus murinus*” to query specimen occurrences documented in the Global Biodiversity Information Facility (GBIF; Edwards 2004). As of November 2015, this query returns some 540 records whose respective years of acquisition range from 1883 to 2011. Looking at Figures 1–3, we can immediately identify an opportunity to improve query granularity and reliability by recognizing taxonomic concept labels and connecting them with RCC-5 articulations (see also Tables 3–5). In particular, the two MSW standards endorse two noncongruent taxonomic concepts whose respective labels share the name component “*Microcebus murinus*.” The earlier 1993.Microcebus_murinus is properly inclusive of (>) the later 2005.Microcebus_murinus.

Suppose that each of the 540 GBIF records was identified explicitly to either the MSW2 or MSW3 primate classification standard. This is often deemed best practice, and many biodiversity data platforms allow contributors to specify the classification scheme used to identity specimens (e.g., Constable *et al.* 2010; Gries *et al.* 2014; Lepage *et al.* 2014). We could then exploit the corresponding set of reasoner-inferred MIR to formulate queries of the following types (see also Remsen 2016). (i) Return all records identified to the name *Microcebus murinus* (optionally, with synonyms or algorithmically matched names). This query corresponds to the current capability of many portals and services (Patterson *et al.* 2010; Boyle *et al.* 2013; Rees 2014b). What follows reaches beyond name-based resolution of occurrence records. (ii) Return all records identified to the concept *Microcebus murinus* sec. Groves (1993) and, alternatively, *Microcebus murinus* sec. Groves (2005). Display the alternative occurrence distribution maps. (iii) Translate all identifications of records to MSW3-endorsed concepts into their corresponding identifications to MSW2-endorsed concepts. This direction of specimen-to-concept identification translation is more facile than the inverse query (i.e., translate MSW2 to MSW3), because the MSW3 classification is more granular (Table 1). Additional criteria such as geographic separation of more narrowly circumscribed entities can aid in achieving such translations (Weakley 2015). (iv) Highlight “problem specimens” that are potentially identifiable to multiple overlapping concepts, given the classification standards used (over time) to carry out identifications. (v) Display records in this target region as identified according to the most, or least, granular concept-level taxonomy or (potentially) set of “composite” concept-level taxonomies. (vi) For *any* set of specimens and associated biological data identified to *any* pair of concepts represented in the MIR (there are 153,111 such pairs in the MIR of the Prim-UC), assess whether the specimens and data can or cannot be integrated. Four out of five articulations—congruence (==), proper inclusion (>), inverse proper inclusion (<), and exclusion (|)—provide either unidirectionally (>, <) or bidirectionally (==, |) actionable information to answer this query. Most problematic are overlapping articulations (><, *N* =49 in the Prim-UC; see Table 3). However, if the Euler subregions that result from input concept overlap are resolved, the integration challenge is simplified to the level of (inverse) proper inclusion (Fig. 9).

We suggest that the above queries and others that leverage multi-taxonomy alignments are needed for the creation of open-ended, taxonomically evolving, and semantically powerful biodiversity data portals (Franz *et al.* 2008). Sound data integration semantics are the precondition for scaling to larger data and time scales. Although trained humans or Natural Language Processing applications (Cui 2012) can examine the information in Figure 2 and perform many of the integration tasks “intuitively,” logic formalizations in the form of RCC-5 articulations have advantages over recurrent, “for-human-minds-only,” name:meaning reconciliation efforts that remain standard practice for original and synthesis projects. These include: enhanced explicitness, clarity, consistency, and above all machine interpretability of evolving taxonomic meanings (Table 3).

The Prim-UC entails 609 species-level concepts, but only 151 instances in which the same species name reliably identifies congruent taxonomic entities across the two MSW editions (Table 4). Biodiversity data environments should have direct access to such information. As we advance further into the networked, open-ended, data-driven age, we should have the ability to perform logic-based integration tasks on taxonomically annotated data flexibly across platforms, and at scales that humans can no longer effectively process. We reiterate, however, that the logic requirement is focused on *how* we express similarities and differences in meaning across taxonomies—better than we are able to based on names alone. Neither the input taxonomies nor the specific articulations are thereby considered products of strict computational logic. The RCC-5 alignment approach remains fundamentally dependent on inferences of taxonomic congruence as advocated by particular human speakers, and on reconciliations of meanings across multiple succeeding taxonomies *as understood by these human experts.* Attribution of human speaker expertise is hard-wired into the approach. Ideally, this interplay of expert input and logic representation can leverage both human and computational strengths toward scalable integration outcomes.

#### Phylogeny assembly platforms

The RCC-5 alignment approach is just as useful for representing and resolving conflict and ambiguity across multiple tree inferences that are synthesized in open-ended phylogeny platforms (e.g., Hinchcliff *et al.* 2015). Even though the Prim-UC contains only ranked concepts, the approach is well suited for aligning informally named (rankless) clade-level concepts (Franz *et al.* 2008, 2015; Franz and Peet 2009). As discussed above (second objection of the preceding section), if coverage is applied to parent concepts that entail incongruent sets of children due to phylogenetic additions and rearrangements, then overlapping articulations will be frequent among the parent concepts (Fig. 4). This kind of multi-phylogeny overlap is challenging to represent with either the Linnaean naming system or phyloreferences (Bryant and Cantino 2002; Dubois 2005).

Consider the occurrences of the name “Cebidae” in the alternative MSW2/MSW3 hierarchies (Figs. 4 and 6, and Supplementary Fig. S3–2 available on Dryad). The alignment depicted in Fig. 6A illustrates the complex articulations of 1993.Cebidae with articulated MSW3 concepts. In particular, the concept 1993.Cebidae has overlapping articulations (><) with 2005.Cebidae and 2005.Pitheciidae, due in part to incongruent assignments of genus-level concepts such as *Callicebus* sec. Groves (1993/2005) or *Callithrix* sec. Groves (1993/2005) to MSW2/MSW3 subfamily- and family-level concepts.

This example illustrates another desirable feature of the RCC-5 alignment approach. Whenever two input concept regions—C_1_ and C_2_—are inferred to overlap, three Euler subregions are created in the alignment: (i) the subregion that is unique to C_1_ (C_1_\C_2_; read: “C_1_, *not* C_2”_); (ii) the subregion that is unique to C_2_ (C_2_ C_1_); and (iii) the subregion to which each input region\partially contributes (C_1_*C_2_; read: “C_1_ *and* C_2_”). None of the resulting subregions are appropriately identified in the input taxonomies, which only contain labels for C_1_ and C_2_ (Franz *et al.* 2015, 2016). Nevertheless, the ability to refer to these subregions is critical for integrating phylogenetically overlapping concept trees.

The Euler/X toolkit command “combined concepts” can resolve Euler subregions resulting from overlap according to this labeling convention (Fig. 9B). The visualization provides valuable insights into the identity of congruent and unique Euler subregions in the MSW2/MSW3 alignment. We can now circumscribe the concept 1993.Cebidae through uniquely identified sets of subordinate regions that variously correspond to the concepts of Groves (2005). Euler subregions with two parents are of special importance for understanding an alignment with overlap, because they represent the congruent C_1_*C_2_ regions in such cases. Accordingly, we observe that the shared subregions 2005.Cebidae*1993.Cebidae and 2005.Pitheciidae*1993.Cebidae are differentially subsumed under either (i) 2005.Pitheciidae and 2005.Cebidae or (ii) 1993.Cebidae. The family-level concepts 2005.Aotidae and 2005.Atelidae are also incongruently assigned to MSW2/MSW3 parent concepts (with unequal ranks). The 2005.Callitrichinae are congruent with precisely that subregion (2005.Cebidae\1993.Cebidae) of the MSW3 classification which is *not* subsumed under 1993.Cebidae. Meanwhile, 2005.Callithrix contains entities—that is, seven acquisition-contingent species-level concepts sec. Groves (2005)—*not* subsumed (2005.Callithrix\1993.Callitrichidae) under 1993.Callitrichidae. An analogous alignment pattern holds for 2005.Callicebus in relation to 1993.Cebidae, that is, the unique subregion 2005.Callicebus\1993.Cebidae entails three acquisition-contingent species-level concepts sec. Groves (2005). Visualizing the reduced containment alignment with input concept overlap (Fig. 9A), and then resolving that overlap in the combined concept alignment (Fig. 9B), can provide an enhanced understanding of taxonomic and congruence in complex cases of phylogenetic conflict.

Dynamic phylogeny assembly platforms are part of the drive to integrate and synthesize our collective, evolving, phylogenetic knowledge (e.g., Thomas 2015). Such platforms will benefit from promoting semantic practices that can precisely resolve phylogenetic congruence across multiple tree hypotheses (Rees 2014a). One reviewer commented (and we concur) that name:meaning analyses of the sort presented in Tables 3–5 can provide metrics for prioritizing new phylogenetic research, by concentrating on perceived clades where prior tree-to-tree congruence is especially low. Using taxonomic concept labels is an effective way to integrate multiple inferences while ensuring attribution of authorship and enabling expert annotations of evolving phylogenetic content. Such contributor accreditation services are difficult to build on top of identifiers such as “*Microcebus murinus*” or “Cebidae,” which in effect act as taxonomic concept *lineage* labels with differential meanings in one or the other synthetic view (Figs. 1, 3, 4, and 6).

## Conclusions

The Prim-UC demonstrates the feasibility of achieving logically consistent, expressive RCC-5 alignments across incongruent classifications with minimally 400 concepts per input taxonomy. More generally, we have shown that the contents of such influential classification standards, and likely of many others, are amenable to formal, logic-based comparison and integration—as long as users are motivated to engage in this translation process. Although the Prim-UC involves larger input taxonomies than previous analyses (Franz *et al.* 2015, 2016; Jansen and Franz 2015), the input concepts are sufficiently well specified and therefore only moderately challenging to articulate. Application or relaxation of the coverage constraint, and optional direct representation of feature-based traits, can yield either member- or property-centric alignments between higher-level concepts. This suggests that the RCC-5 alignment approach is suited to align a wide range of taxonomic and phylogenetic products.

Not surprisingly, our understanding of primate systematics has continued to evolve since the third MSW edition (e.g., Marsh 2014; Pozzi *et al.* 2014; Rylands and Mittermeier 2014). New, and in some instances strongly conflicting inferences are sure to find their way into future, heavily used classification standards for this significant group. Such inferences are bound to produce further name:meaning dissociations in relation to currently endorsed standards. If nearly one in three primate names can no longer play the same semantic role in an interval of only 12 years of systematic advancement, there is an opportunity to express and resolve such conflicts. The RCC-5 alignment approach can counteract the potentially cumulative loss of resolution across succeeding, synthetic classifications. Keeping taxonomic reference backward- and forward-compatible is necessary for systematics to play its long-term, large-scale integrative role in the comparative biological domain.

How far might the new semantics reach? In the widest sense, RCC-5 based alignments are applicable to any set of systematic or otherwise hierarchically arranged data whose identifiers (names) are subject to semantic modulation over time (Wang *et al.* 2011). Hence the approach is also suited to align evolving *character* concepts, or evolving assignments of *specimens* to either taxonomic concepts or characters (Fig. 10; see also Pullan *et al.* 2000; Rieppel 2007; Franz 2014). However, promoting formalized, temporally dynamic representations of similarities and differences of specimen/character/concept assignments will require substantive realignments of virtual biodiversity platforms and data annotation practices (Koperski *et al.* 2000; Kennedy *et al.* 2005; Lepage *et al.* 2014; Borsch *et al.* 2015; Weakley 2015). At each step, the benefits and costs of achieving more granular semantic resolution should be reassessed. Whether the trade-offs are favorable will likely depend on the resolution demands by humans and/or machines to achieve particular inference objectives. We are optimistic that greater accessibility of logic-based alignment tools will clarify their value for integrating the growing body of systematic knowledge in next-generation data platforms.

## Supplementary Material

Supplementary material can be found in the Dryad data repository at http://dx.doi.org/10.5061/dryad.6jg71.
